# Long-term depletion of KIR3DL01+ NK cells by adeno-associated viral-vectored antibody delivery alters chronic SIV infection

**DOI:** 10.1371/journal.ppat.1014508

**Published:** 2026-08-05

**Authors:** Kjell Sandstrom, Grace N. Hagedorn, Sean C. Robinson, Julia E. Katz, Trent M. Prall, Roger W. Wiseman, Jun Xie, Matthew R. Gardner, Andrea M. Weiler, Grace A. VanSleet, Sydney M. Stroschein, José L. Sanchez Hernandez, Amy W. Moy, Jeffrey D. Lifson, Saverio V. Capuano III, Alessandro Poggi, Guangping Gao, David H. O’Connor, Thomas C. Friedrich, Vadim A. Klenchin, David T. Evans

**Affiliations:** 1 Department of Pathology and Laboratory Medicine, University of Wisconsin-Madison, Madison, Wisconsin, United States of America; 2 Department of Genetics and Cellular Medicine, Horae Gene Therapy Center, University of Massachusetts Chan Medical School, Worcester, Massachusetts, United States of America; 3 Department of Medicine, Division of Infectious Diseases, Emory University, Atlanta, GeorgiaUnited States of America; 4 Division of Microbiology and Immunology, Emory National Primate Research Center, Emory University, Atlanta, GeorgiaUnited States of America; 5 Wisconsin National Primate Research Center, University of Wisconsin-Madison, Madison, Wisconsin, United States of America; 6 AIDS and Cancer Virus Program, Frederick National Laboratory for Cancer Research, Frederick, Maryland, United States of America; 7 Molecular Oncology and Angiogenesis Unit, IRCCS Ospedale Policlinico San Martino, Genoa, Italy; University of Illinois at Chicago College of Medicine, UNITED STATES OF AMERICA

## Abstract

Natural killer (NK) cells are key innate effectors during antiviral immune responses, with their activity regulated in part by interactions between polymorphic killer-cell immunoglobulin-like receptors (KIRs) on NK cells and their major histocompatibility complex class I (MHC I) ligands on target cells. In human immunodeficiency virus (HIV) infection, certain *KIR* and *MHC I* allelic combinations are associated with enhanced viral control and delayed disease progression. To interrogate the contribution of a common KIR+ NK cell subset to the immune response to simian immunodeficiency virus (SIV) infection of rhesus macaques, we depleted KIR3DL01+ cells using an adeno-associated virus (AAV) vector encoding a KIR3DL01-reactive monoclonal antibody. AAV delivery resulted in high and durable antibody expression with minimal anti-drug antibody responses, leading to sustained depletion of KIR3DL01+ NK cells from blood, lymph nodes, and gut-associated lymphoid tissue for more than nine months. Following intrarectal SIV challenge, there was no difference in peak viremia between the two groups. However, KIR3DL01-depleted animals exhibited a modest increase in chronic viremia, reaching statistical significance at multiple timepoints relative to KIR3DL01+ controls. Phenotypic analysis revealed ongoing NK cell maturation in peripheral blood and lymphoid tissue, accompanied by increased expression of activation and proliferation markers during acute and early chronic infection. An expansion of NKG2D+ NK cells was also observed in chronic SIV infection. These findings suggest that KIR3DL01+ NK cells contribute to the inhibition of SIV replication during chronic infection. Moreover, they demonstrate the feasibility of AAV-vectored antibody delivery for long-term, perhaps indefinite depletion of a lymphocyte subset in a nonhuman primate model.

## Author summary

Natural killer (NK) cells are an important early defense against viral infections, which rely on diverse surface receptors to distinguish virus-infected cells from healthy cells. Among these are the highly polymorphic killer-cell immunoglobulin-like receptors (KIRs). However, the role of individual KIR-expressing NK cell subsets during infection has been difficult to determine. We therefore utilized an adeno-associated virus (AAV) gene delivery system to selectively deplete a specific NK cell subset in rhesus macaques, an essential animal model for HIV/AIDS research. This approach resulted in long-term production of an antibody targeting the KIR3DL01 receptor, leading to rapid and sustained elimination of KIR3DL01+ NK cells in blood and tissues for more than nine months. When these animals were infected with simian immunodeficiency virus (SIV), a close relative of HIV, KIR3DL01-depleted animals showed a modest increase in viral loads during chronic infection compared to non-depleted KIR3DL01+ control animals. These results suggest that KIR3DL01+ NK cells help to limit SIV replication. This study also highlights the feasibility of using AAV delivered antibodies to deplete specific immune cell populations to study their contribution to antiviral immunity.

## Introduction

Natural killer (NK) cells are innate immune cells that can detect and kill infected or malignant target cells without prior antigenic stimulation. Their cytotoxic and cytokine-producing activity is regulated through the integration of signals from diverse activating and inhibitory receptors [1–3]. Among these, the polymorphic killer-cell immunoglobulin-like receptors (KIRs) play a central role in modulating NK cell activity through interactions with major histocompatibility complex class I (MHC I) molecules [4–6]. KIRs possess two to three immunoglobulin-like domains with long or short cytoplasmic tails, defining them as inhibitory (KIR2DL or KIR3DL) or activating (KIR2DS or KIR3DS) receptors [7]. Inhibitory KIRs contain immunoreceptor tyrosine-based inhibitory motifs (ITIMs) in their cytoplasmic tails, which recruit phosphatases upon ligand engagement to dampen NK cell activation [8,9]. In contrast, activating KIRs associate with adaptor proteins such as DAP12, CD3ζ, and FcεRIγ, which contain immunoreceptor tyrosine-based activating motifs (ITAMs) that transduce activating signals [10,11]. Engagement of inhibitory KIRs by MHC I ligands prevents NK cell responses against healthy cells [4]. In virally infected or transformed cells, disruption of MHC I surface expression diminishes inhibitory signaling, leading to NK cell activation [12–17].

In humans and nonhuman primates, *KIR* genes are located within the leukocyte receptor complex on chromosome 19 [18–22]. Humans typically encode 7–12 functional *KIR* genes organized into haplotypes differing in specific gene combinations [18,19]. In rhesus macaques, the *KIR* genes are similarly expanded and highly polymorphic. Although haplotype organization is less clearly defined, individual rhesus macaques contain on average 12 distinct *KIR* genes per animal [20,22]. KIRs are expressed in a stochastic and variegated manner, which generates NK cell repertoires with different combinations of activating and inhibitory KIRs on any given cell. This diversity is generated during maturation in the bone marrow, producing functionally heterogeneous NK cell subsets [23–25]. During development, engagement of inhibitory KIRs with cognate MHC I ligands “licenses” or “educates” NK cells, conferring enhanced functional responsiveness when inhibitory interactions are subsequently lost or when activating receptor ligands are encountered [6,26,27].

Given their central role in regulating NK cell responses, specific combinations of *KIR* and *MHC I* alleles have been implicated in the control of viral infections, including human and simian immunodeficiency viruses (HIV and SIV). In people living with HIV, *KIR3DL1* or *KIR3DS1* together with high-affinity *HLA-Bw4* alleles encoding isoleucine at position 80 (I80) are associated with delayed progression to AIDS [28,29]. Strong inhibitory interactions between KIR3DL1 and HLA-Bw4 I80 ligands calibrate KIR3DL1+ NK cells to a highly licensed state [29,30], enhancing their ability to detect HIV Nef-mediated downregulation of HLA-B [15]. In SIV-infected rhesus macaques, studies examining associations between *KIR* polymorphisms and disease progression have been limited due to the extensive diversity of *KIR* genes, the rapid evolutionary divergence between species, and the lack of KIR-specific reagents in this nonhuman primate model. However, while not a direct ortholog of human *KIR3DL1*, rhesus macaque *KIR3DL01* similarly binds Mamu-B allotypes encoding a Bw4 motif [31]. *KIR3DL01* is present in approximately 80% of rhesus macaques of Indian ancestry and exhibits substantial allotypic polymorphism [20,22]. Therefore, targeting this subset enables direct *in vivo* evaluation of Bw4-licensed NK cells during immunodeficiency virus infection.

To determine the contribution of KIR3DL01+ NK cells to the control of SIV infection, we selectively depleted this subset using an adeno-associated virus (AAV) vector encoding a KIR3DL01-specific antibody prior to SIV challenge [31]. Six KIR3DL01+ animals received AAV vectors encoding the KIR3DL01-depleting antibody and six mock-treated KIR3DL01+ animals served as controls. Sustained depletion of KIR3DL01+ NK cells was achieved in blood, gut-associated lymphoid tissue (GALT), and lymph nodes for over 40 weeks. Following SIV challenge, the prolonged loss of this specific NK cell subset resulted in a modest increase in average chronic phase viremia, with viral loads significantly elevated at multiple timepoints during chronic infection.

## Results

### KIR and MHC I characterization of study animals

To investigate the functional role of KIR3DL01+ NK cells in SIV control, we aimed to selectively deplete this population prior to SIV infection. Prospective study animals were *MHC I* genotyped to exclude *MHC I* alleles associated with spontaneous control of SIV infection (Mamu-B*008 and -B*017) (S1 Table) [32,33]. Five AAV-treated and four control animals expressed *Mamu-B* alleles previously identified as ligands of KIR3DL01 (S1 Table, bold) [31]. Furthermore, all study animals encoded at least two *Mamu-B* alleles containing a Bw4 motif. KIR3DL01+ rhesus macaques were initially identified by staining PBMCs with the KIR3DL01-reactive NKVFS1 antibody [31], and subsequently confirmed by *KIR* genotyping (S2 Table). To ensure the specificity of the NKVFS1 antibody and rule out off-target binding, Jurkat cells transduced with FLAG-tagged rhesus macaque KIR constructs were stained with NKVFS1, confirming selective binding to KIR3DL01-expressing cells and no cross-reactivity with 24 other KIR gene products (S1 Fig). Because the NKVFS1 antibody only binds KIR3DL01 allotypes containing an aspartic acid (D233) but not a histidine at (H233) at position 233 [31], all AAV-treated animals were confirmed to be D233 homozygous to guarantee complete depletion. However, three control animals were D233/H233 heterozygous, meaning NKVFS1 staining may underestimate KIR3DL01+ NK cell frequencies in these animals (S3 Table).

Four animals in each group also exhibited detectable staining of NK cells with Mamu-A1*002 Gag 71–79 GY9 tetramer, which identifies *KIR3DL05*+ animals as previously described [34–36]. In one A1*002 tetramer+ animal (rhbo12), a *KIR3DL05* allele was not detected by *KIR* cDNA sequencing (S2 Table), even though the intensity of Mamu-A1*002 tetramer staining was comparable to other *KIR3DL05*+ macaques (S2 Fig), and none of the other *KIR* alleles identified in rhbo12 have been observed to interact with Mamu-A1*002 [34,37]. This suggests the presence of a *KIR3DL05* allele in rhbo12 that was not captured by cDNA primer amplification.

### Phenotyping of KIR-defined NK cell subsets

Prior to AAV administration, PBMCs were stained with an antibody panel to evaluate NK cell phenotypes across KIR3DL01+ and KIR3DL05+ subsets (S2 Fig). In rhesus macaques, NK cell maturation progresses from less-differentiated, cytokine-producing CD16-CD56+, through an intermediate double-negative (DN) stage, to highly differentiated, cytolytic CD16+ CD56- cells [38–40]. KIR3DL01+ single-positive NK cells (KIR3DL01+ KIR3DL05-) demonstrated a trend towards a more mature CD16+ CD56- phenotype compared to KIR3DL01- KIR3DL05- cells (p = 0.0505) (S3 Fig). Furthermore, the KIR3DL01+ KIR3DL05- subset exhibited lower frequencies of both immature CD16-CD56+ and intermediate CD16-CD56- NK cells relative to KIR3DL01-KIR3DL05-. Interestingly, the KIR3DL05+ KIR3DL01- subset contained a significantly higher frequency of NKG2D+ cells compared to the KIR3DL01+ subset, alongside a trend towards elevated expression relative to the KIR3DL01-KIR3DL05- population (S3 Fig).

### AAV-administered antibody expression

**Fig 1:**
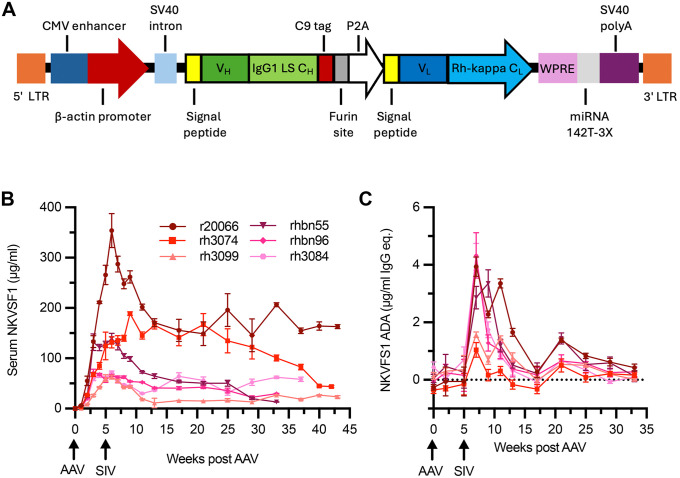
Stable AAV9-mediated NKVFS1 expression yields high antibody levels with minimal anti-drug antibody responses. **(A)** Diagram of NKVFS1-AAV9 expression construct. Expression was driven by a CMV enhancer and chicken β-actin promoter. Heavy and light chains were linked by a P2A sequence and furin cleavage site, including LS mutations (M428L/N434S) for extended half-life, a rhodopsin tag (C9) for ELISA detection, and three miR-142T sites to restrict expression in antigen-presenting cells. **(B)** Serum NKVFS1 concentration was quantified by ELISA using plates coated with an anti-rhodopsin antibody (1D4). Error bars indicate the standard deviation of at least 3 technical replicates. **(C)** Anti-drug antibody responses were assessed by purifying and biotinylating total serum IgG and quantifying the amount of biotinylated IgG bound to immobilized NKVFS1 by a modified ELISA. Error bars indicate the standard deviation of at least 3 technical replicates.

For sustained depletion of KIR3DL01+ NK cells, we generated an AAV vector encoding a rhesus macaque IgG version of the KIR3DL01-binding NKVFS1 antibody. Variable heavy and light chain sequences for NKVFS1 were cloned in frame with sequences encoding rhesus macaque IgG1 constant regions. The “simianized” NKVFS1 was cloned into an AAV expression construct, in which antibody expression is driven by a CMV enhancer/chicken β-actin promoter, with heavy and light chains separated by a P2A ribosomal skip sequence and furin cleavage site for the removal of P2A-encoding residues (Fig 1A). LS substitutions (M428L and N434S) were introduced into the heavy chain to enhance neonatal Fc receptor (FcRn) binding and extend serum half-life of the antibody [41,42], and a rhodopsin tag (C9) was added to the C-terminus of the heavy chain for quantification by ELISA. To minimize the immune response against the transgene, the AAV expression construct incorporated three micro-RNA binding sites (miR-142T) to suppress NKVFS1 expression in professional antigen presenting cells (APCs) (Fig 1A) [43,44]. The NKVFS1 expression cassette was packaged in an AAV serotype 9 capsid (AAV9) for *in vivo* delivery. All animals were pre-screened for neutralizing antibodies against the AAV9 capsid to exclude seropositive animals from the AAV treatment group (S3 Table).

Five weeks prior to SIV challenge, six KIR3DL01+ rhesus macaques received the NKVFS1-AAV9 vector by intramuscular (IM) injection at a dose of 3.0x10^12^ genome copies per kilogram (gc/kg) and six KIR3DL01+ control animals received IM injections of phosphate buffered saline (PBS). Concentrations of NKVFS1 in serum varied among the animals treated with NKVFS1-AAV9. Antibody expression peaked at six weeks post-AAV (62–354 µg/ml, median: 102 µg/ml) and stabilized by week 13 (11–171 µg/ml, median: 65 µg/ml) (Fig 1B). Anti-drug antibody (ADA) titers were minimal throughout the study. ADA titers peaked at seven weeks post-AAV (1.04-4.33 µg/ml, median: 3.4 µg/ml IgG equivalent) and declined throughout the remainder of the study (median: 0.5 µg/ml IgG equivalent) (Fig 1C). The timing of peak ADA responses coincided with peak NKVFS1 expression at weeks 6–7 post-AAV, and animals with higher circulating NKVFS1 levels exhibited correspondingly higher, though still minimal, ADA responses (Fig 1B and 1C).

### Depletion of KIR3DL01+ NK cells and CD8 T cells in blood and tissues

PBMCs were stained with separate antibody panels to assess NK cell phenotypes (S2 Fig), T cell phenotypes (S4 Fig), and KIR3DL01 depletion (S5 Fig). To quantify total cell populations, absolute cell counts were measured by whole blood staining with antibodies against lineage-specific markers and calculated using a bead-based standard (S6 Fig) and multiplied by the cell frequencies obtained from the phenotyping panels. NK cells were defined as CD3- CD8α+NKG2AC+ and T cells were defined as CD3+.

**Fig 2:**
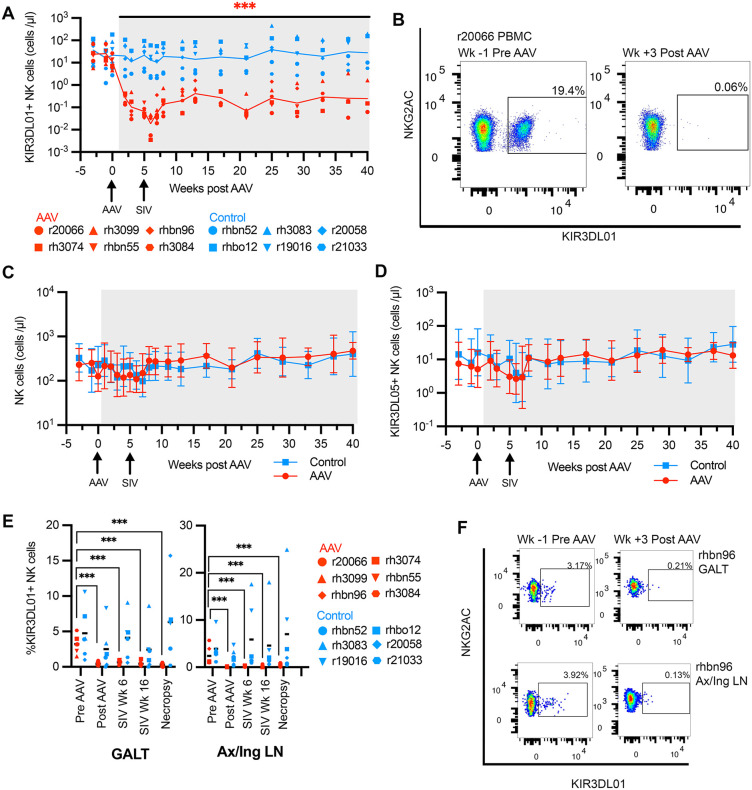
AAV delivered NKVFS1 induces complete and durable depletion of KIR3DL01+ NK cells in blood and tissues. Throughout the figure, control animals are shown in blue and AAV-treated animals in red. **(A)** Longitudinal depletion of KIR3DL01+ NK cells in peripheral blood. Lines indicate geometric means of log_10_-transformed cell counts per group. Asterisks indicate significant differences between the AAV-treated and control groups by linear mixed-effects modeling (*** p < 0.001). **(B)** Representative flow cytometry plots showing KIR3DL01+ NK cells in blood before and after AAV administration. **(C)** Longitudinal changes in total NK cell counts in blood. Lines indicate geometric means ± SD of the log_10_-transformed number of cell counts per group. **(D)** Longitudinal changes in KIR3DL05+ NK cell counts in blood. Lines indicate geometric means ± SD of the log_10_-transformed number of cell counts per group. Absolute NK cell counts (CD3/20-, NKG2AC/CD8α+) were determined by Trucount whole-blood staining. Absolute counts of KIR3DL01+ and KIR3DL05+ NK cells were extrapolated by multiplying their frequency within total NK cells (from NK cell phenotyping panel) by total NK cell counts. The shaded region indicates post-AAV timepoints. **(E)** Longitudinal changes in the frequency of KIR3DL01+ NK cells in GALT and LN tissue. Data are expressed as percentages of total NK cells. Asterisks indicate significance relative to the pre-AAV timepoint by linear mixed-effects modeling (*** p < 0.001). **(F)** Representative staining of KIR3DL01+ NK cells in tissues before and after AAV administration.

Prior to AAV administration (weeks -3, -1, and 0 pre-AAV), KIR3DL01 expression on blood NK cells was comparable between the groups, averaging 15.2% in the AAV-treatment group and 15.9% in control animals (S3 Table). KIR3DL01+ NK cell depletion in peripheral blood was evident as early as one week following AAV administration, with serum antibody concentrations as low as 2.9 µg/ml in one animal (rhbn55) (Figs 1B, 2A). The AAV delivered NKVFS1 antibody incorporates a rhodopsin tag, allowing direct assessment of its binding to KIR3DL01+ cells (Fig 1A). Low level KIR3DL01+ rhodopsin+ co-staining was detected in two animals at one week post-AAV and was absent at all later timepoints (S7 Fig). This transient co-staining indicates that the loss of KIR3DL01 staining could not be explained simply by receptor blockade. Complete KIR3DL01+ lymphocyte depletion was achieved in all six animals that received the NKVFS1-AAV9 vector by two weeks post-AAV and continued through 40 weeks post-AAV, with KIR3DL01+ NK cells declining from a pre-AAV mean of 27.5 cells/µl (range: 6.35 – 55.02 cells/µl) to a post-AAV mean of 0.3 cells/µl (range: 0.08 – 0.79 cells/µl) in peripheral blood (Fig 2A and 2B). Despite robust and persistent depletion of KIR3DL01+ NK cells, total NK cell frequencies were unchanged relative to controls after AAV-treatment (Fig 2C) and no changes in KIR3DL05+ NK cell frequencies were observed (Fig 2D).

**Fig 3:**
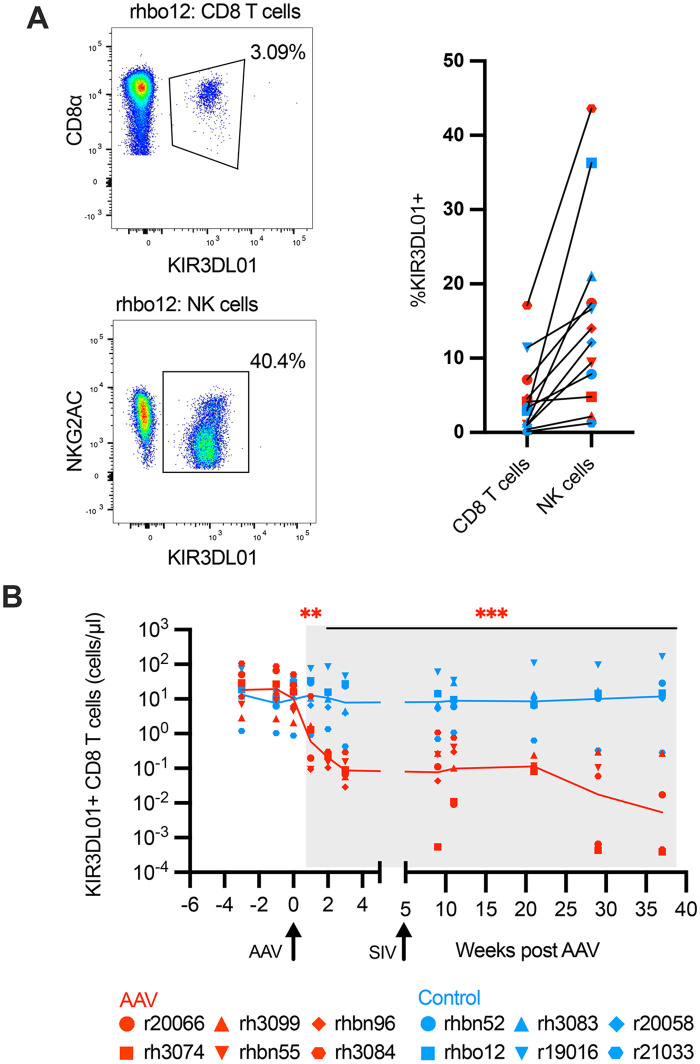
Depletion of KIR3DL01+ CD8 T cells. **(A)** Representative flow plot showing KIR3DL01 expression on CD8 T cells and NK cells. Pre-AAV KIR3DL01 expression on CD8 T cells and NK cells averaged and matched per animal (Week -3, -2, and 0 pre-AAV). **(B)** Depletion of KIR3DL01+ CD8 T cells in blood. Lines indicate geometric means of log_10_-transformed cell counts per group (control: blue and AAV: red). Absolute counts were determined by multiplying the frequency of KIR3DL01+ CD8 T cells by total CD8 T cells counts at each timepoint. The shaded region indicates post-AAV timepoints. Asterisks indicate significant differences between AAV-treated and control group by linear mixed-effects modeling (** p < 0.01, *** p < 0.001).

To evaluate depletion in tissues, lymph node (LN) and colon biopsies were collected, and stained with an NK cell phenotyping antibody panel. Overall, NK cells in tissues expressed lower levels of KIRs compared to blood. Pre-AAV, average KIR3DL01 expression on NK cells was 15.5% in peripheral blood (S3 Table), 3.1% in LN, and 4.0% in GALT (Fig 2E). Consistent with our findings in blood, KIR3DL01+ NK cells were depleted in both LN (mean: 0.10%, range: 0 - 0.16%) and GALT (mean: 0.42%, range: 0.09 - 0.9%) by 3 weeks post-AAV administration (Fig 2E and 2F). This depletion persisted through the course of SIV infection until necropsy at more than 40 weeks post-AAV administration for both LN (mean: 0.42%, range: 0 - 0.91%) and GALT (mean: 0.32%, range: 0 - 0.62%) (Fig 2E, 2F). KIR3DL01 expression was also observed on a subset of CD8 T cells (4.5% pre-AAV mean), albeit at a lower frequency compared with NK cells (Fig 3A). KIR3DL01+ CD8 T cells were depleted as early as one week post-AAV administration and remained suppressed throughout the 40-week study period, with peripheral blood counts declining from a pre-AAV mean of 28.9 cells/µl (range: 2.6 – 82.1 cells/µl) to a post-AAV mean of 0.5 cells/µl (range: 0.1 – 1.9 cells/µl) (Fig 3B). Despite the loss of this specific KIR+ CD8 T cell subset, longitudinal analysis revealed no significant differences in the frequencies of total T cells, total CD8 T cells, or effector memory CD8 T cells between the AAV-treated and control groups (S8A-S8C Fig).

### Impact of KIR3DL01+ NK cell depletion on viral loads and memory CD4 T cells

**Fig 4:**
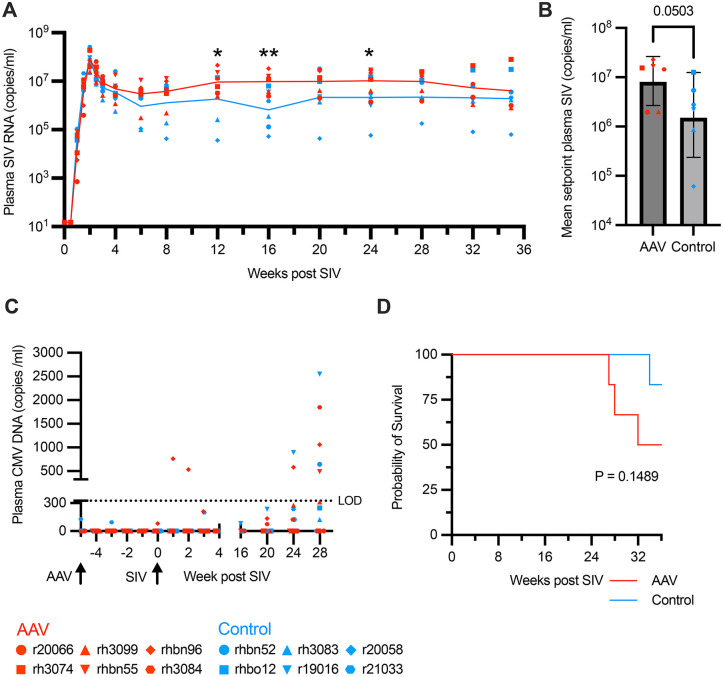
Effects of KIR3DL01+ NK cell depletion on viral loads. Throughout the figure, control animals are shown in blue and AAV-treated animals in red. **(A)** Plasma SIV viral loads following intrarectal SIVmac239 challenge (3,000 TCID50) in AAV-treated and control animals. Viral RNA was quantified by qRT-PCR with a detection limit of 15 copies/ml. Lines indicate geometric means of log_10_-transformed SIV copies/ml per group. Asterisks indicate significant differences between the AAV and control groups by linear mixed-effects modeling (* p < 0.05, ** p < 0.01). **(B)** Comparison of averaged chronic phase SIV viral load. Data represent mean and standard deviation of log_10_-transformed plasma viral load averaged per animal from week 8 to week 35 post-infection. Statistical significance determined by unpaired t-test (p = 0.0503). **(C)** Reactivation of endemic rhCMV was assessed in plasma by real-time PCR targeting rhCMV UL54 with a limit of detection of 325 DNA copies/ml. **(D)** Kaplan-Meier survival analysis. Necropsies prior to week 36 were performed based on clinical criteria determined by veterinary staff. Statistical significance was evaluated using the Gehan-Breslow-Wilcoxon test.

Five weeks after AAV administration, KIR3DL01-depleted and control animals were challenged intrarectally with SIVmac239 (3,000 TCID50), a pathogenic infectious molecular clone that is well-adapted for replication in rhesus macaques. Peak plasma viral loads at two weeks post-infection (PI) did not differ significantly between groups (Fig 4A). However, during chronic infection, the AAV-treated group exhibited modestly higher viral loads reaching significance at weeks 12 (p = 0.044), 16 (p = 0.0014), and 24 (p = 0.0498) PI by linear mixed-effects modeling (Fig 4A). Accordingly, average chronic phase viremia (weeks 8–35 PI) was 0.70 log_10_ higher in the AAV-treated group relative to controls, closely approaching statistical significance (p = 0.0503) (Fig 4B). The AAV-treated animals also exhibited a trend towards lower memory CD4 T cell counts during chronic infection (mean: 66.5 cells/µl) compared with controls (mean: 114.9 cells/µl), although this difference was not statistically significant (S8E Fig).

NK cells play a well-established role in controlling human cytomegalovirus (CMV) infection [45–47]. Given the high prevalence of rhesus CMV (rhCMV) among captive-bred rhesus macaques [48], we assessed whether KIR3DL01+ NK cell depletion triggered rhCMV reactivation. No reactivation of rhCMV was detected by qPCR immediately following AAV administration (Fig 4C). Following SIV infection, rhCMV reactivation was sporadic. Reactivation of rhCMV occurred in one animal during acute infection, and in 3 of 6 AAV-treated and 2 of 6 control animals during chronic SIV infection (weeks 24–28 PI), however, this difference was not statistically significant (Fig 4C). Furthermore, while a higher proportion of AAV-treated animals (3 of 6) required early euthanasia due to AIDS-related complications compared to controls (1 of 6), this difference did not reach statistical significance (Fig 4D).

### Phenotypic changes in peripheral blood NK cells

**Fig 5:**
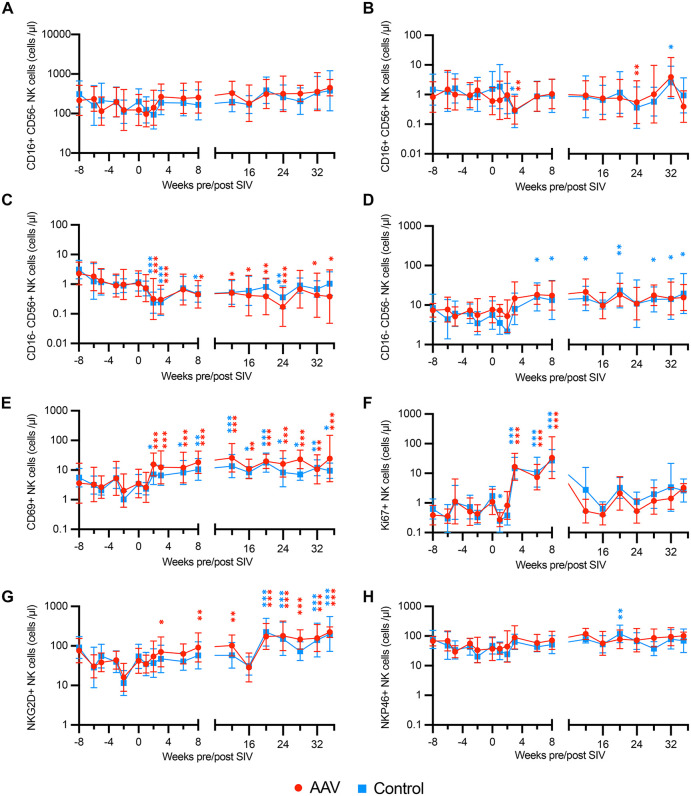
Phenotypic changes in peripheral blood NK cells during SIV infection. Longitudinal changes in absolute counts of CD16+ CD56- **(A)**, CD16+ CD56+ **(B)**, CD16-CD56+ **(C)**, CD16-CD56- **(D)**, CD69+ **(E)**, Ki67+ **(F)**, NKG2D+ **(G)**, and NKp46+ **(H)** NK cells. Absolute counts were calculated by multiplying each marker’s frequency within total NK cells by total NK cell counts determined by Trucount whole-blood staining. Lines indicate geometric means ± SD of log_10_-transformed cell counts per group (control: blue and AAV: red). Asterisks indicate significant differences relative to the averaged pre-SIV baseline (weeks -3 through 0) by linear mixed-effects modeling (* p < 0.05, ** p < 0.01, *** p < 0.001) color-coded per group.

To evaluate phenotypic changes in NK cells during SIV infection, we quantified specific subsets based on markers of maturation (CD16 and CD56), activation (CD69), proliferation (Ki67), and natural cytotoxicity (NKG2D and NKp46) (S2 and S6 Figs). Total NK cell numbers did not significantly change in either group during SIV infection relative to pre-infection baselines (Fig 2C). Assessing maturation states, both groups exhibited a significant decline in CD16-CD56+ NK cells during acute infection (weeks 2–3 PI), with the AAV-treated group maintaining sustained lower levels throughout chronic infection (Fig 5C). In contrast, the control group displayed a sustained elevation of CD16-CD56- DN NK cells during chronic infection (weeks 6–35 PI) (Fig 5D). Following SIV infection, both groups exhibited a significant increase in CD69+ NK cells relative to pre-infection levels, beginning at week 2 (peak viremia) and remaining elevated through 35 weeks PI (Fig 5E). This increase in activation was followed by a transient expansion of Ki67+ NK cells, evident from week 3 and sustained through week 8 PI (Fig 5F). Additionally, the frequency of NKG2D+ NK cells increased during late chronic infection (weeks 20–35 PI) in both groups (Fig 5G).

### Phenotypic changes in tissue NK cell subsets

**Fig 6:**
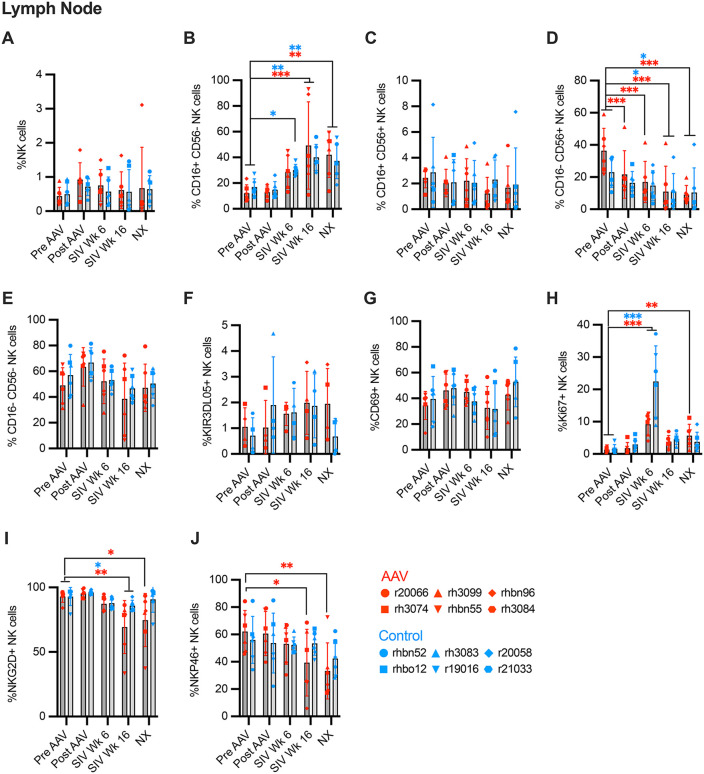
Phenotypic changes in lymph node NK cells. Longitudinal changes in the percentage of NK cells (CD3-NKG2AC+CD8α+) of CD45+ leukocytes **(A)**, and percent of CD16+ CD56- **(B)**, CD16+ CD56+ **(C)**, CD16-CD56+ **(D)**, CD16-CD56- **(E)**, KIR3DL05+ **(F),** CD69+ **(G)**, Ki67+ **(H)**, NKG2D+ **(I)**, and NKp46+ **(J)** of NK cells in LN tissue. Marker expression was determined from NK cell phenotyping flow cytometry panel. Bars represent group means ± SD color-coded per group (control: blue and AAV: red). Asterisks indicate significant differences relative to the pre-AAV timepoint by linear mixed-effects modeling (* p < 0.05, ** p < 0.01, *** p < 0.001) color-coded per group.

**Fig 7:**
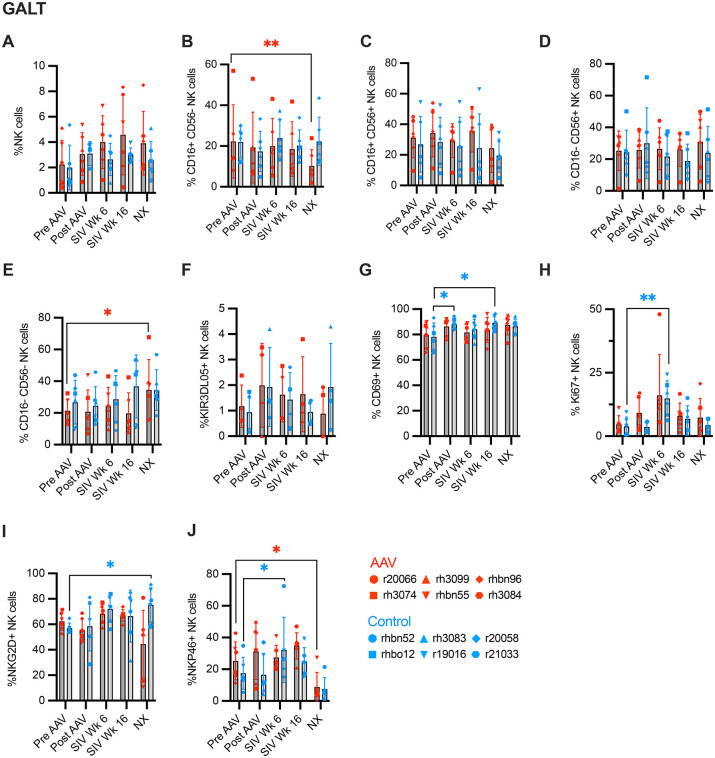
Phenotypic changes in GALT NK cells. Longitudinal changes in the percentage of NK cells (CD3-NKG2AC+CD8α+) of CD45+ leukocytes **(A)**, and percent of CD16+ CD56- **(B)**, CD16+ CD56+ **(C)**, CD16-CD56+ **(D)**, CD16-CD56- **(E)**, KIR3DL05+ **(F),** CD69+ **(G)**, Ki67+ **(H)**, NKG2D+ **(I)**, and NKp46+ **(J)** of NK cells in GALT. Marker expression was determined from NK cell phenotyping flow cytometry panel. Bars represent group means ± SD color-coded per group (control: blue and AAV: red). Asterisks indicate significant differences relative to the pre-AAV timepoint by linear mixed-effects modeling (* p < 0.05, ** p < 0.01, *** p < 0.001) color-coded per group.

To evaluate phenotypic changes in tissue NK cells during SIV infection, we analyzed lymphocytes isolated from lymph nodes and GALT by flow cytometry. The proportion of NK cells among total CD45+ leukocytes did not change significantly in either LN (Fig 6A) or GALT (Fig 7A) over the course of SIV infection. Consistent with prior reports, baseline NK cell subset distributions differed between blood and tissues [38,49,50]. Prior to SIV infection, peripheral blood NK cells were predominantly mature CD16+ CD56-. In contrast, LNs were enriched for less-differentiated CD16-CD56+ and intermediate CD16-CD56- DN NK cell subsets, while the GALT exhibited a more balanced distribution of maturation states (Figs 6B-6E and 7B-7E).

Following SIV infection, NK cells within LNs exhibited a progressive increase in the frequency of mature CD16+ CD56- cells, accompanied by a corresponding decrease in CD16-CD56+ NK cells in both groups (Fig 6B, 6D). This indicates a shift in the LN NK cell compartment towards a more mature and cytolytic phenotype following the onset of viremia [50,51]. In contrast, aside from minor changes observed in tissues collected at necropsy, no notable alterations in NK cell maturation status were detected in GALT over the course of infection (Fig 7B-7E). Reflecting their tissue localization, NK cells in both LN and GALT maintained higher CD69 expression than peripheral blood NK cells (Figs 6G and 7G) [49,52]. In LNs, Ki67 expression increased on NK cells in both groups at week 6 PI relative to pre-SIV baseline, and AAV-treated animals also displayed a significant increase in Ki67 expression at necropsy (Fig 6H). In GALT, Ki67 upregulation at week 6 was only observed in the control animals (Fig 7H). Notably, the timing of Ki67 upregulation in LN and GALT mirrors the transient expansion of Ki67+ NK cells detected in peripheral blood (Fig 5F). In contrast to peripheral blood, LN NK cells exhibited a modest but significant decline in NKG2D expression during chronic SIV infection (Week 16 and necropsy), primarily in the AAV-treated group (Fig 6I). Furthermore, the AAV-treated group demonstrated a modest but significant decrease in LN NKp46+ NK cells from week 16 through necropsy (Fig 6J).

## Discussion

To assess the *in vivo* contribution of KIR3DL01+ NK cells to the control of SIVmac239 infection, we utilized AAV-mediated monoclonal antibody delivery to selectively deplete this immune cell subset in rhesus macaques. Our approach achieved rapid and durable depletion of KIR3DL01+ NK cells, resulting in nearly a complete loss of this cell population in blood, lymph nodes, and GALT for more than nine months. Although KIR3DL01+ NK cell depletion did not alter peak viral loads, it led to a modest increase in chronic phase viremia, with statistically significant increases at multiple timepoints, suggesting a role for this NK cell subset in the reduction of SIV replication. Beyond the biological impact, this approach represents the first long-term depletion of a specific immune cell subset in a nonhuman primate model using AAV-vectored antibodies.

Despite the sustained loss of KIR3DL01+ NK cells, total NK cell levels remained stable and no expansion of KIR3DL05+ NK cells was observed, indicating that depletion was highly specific and did not trigger measurable homeostatic proliferation of other NK cell populations. Although KIR3DL01+ NK cells constitute only ~15% of circulating NK cells, the robust antibody expression achieved with AAV delivery (median: 65 µg/ml) suggests that this platform may be capable of depleting more abundant immune cell populations. Consistent with this, a prior study of B cell depletion in cynomolgus macaques by passive administration of rituximab reported transient depletion at serum antibody concentrations of approximately 70 µg/ml [53], which is comparable to antibody levels achieved here and in other AAV-mediated antibody studies [54,55]. While AAV delivered antibodies have been applied for therapeutic and prophylactic studies [54,56,57], sustained immune cell depletion using this approach has so far only been reported once previously in a mouse model [58]. Together, these findings highlight AAV antibody delivery as a strategy for achieving long-term depletion of cell populations, enabling *in vivo* evaluation of the contributions of specific immune cell subsets to antiviral immunity.

Although the vectored antibody contained a rhesus macaque IgG1 constant region to enable Fc-mediated depletion of KIR3DL01+ NK cells, it retained the original murine NKVFS1 variable domains, raising the possibility of immunogenicity and the development of ADA responses. Prior studies delivering simianized human broadly neutralizing antibodies (bNAbs) against the HIV envelope glycoprotein in rhesus macaques have engendered robust ADA responses that ablated antibody expression [59,60]. Even antibodies derived from rhesus macaques have elicited ADAs in this model [54,55,57]. Surprisingly, we observed only minimal ADA responses to NKVFS1 despite the presence of murine variable regions. Previous studies have shown that the degree of somatic hypermutation within the variable regions is a major determinant of ADA responses [61,62]. In this case, murine variable regions with minimal affinity maturation may be better tolerated. Additional factors, including the incorporation of miR-142T sites that restrict antibody expression in APCs [43], as well as sustained high-level antigen exposure possibly inducing high-zone tolerance [63,64], may have contributed to limited ADAs in this study.

Following complete depletion of KIR3DL01+ NK cells, we observed no significant difference in peak SIV RNA levels at day 14 PI, indicating that the loss of this NK cell subset does not substantially impact acute viral replication. However, during chronic infection, KIR3DL01-depleted animals exhibited significantly elevated plasma viral loads at multiple timepoints by linear mixed-effects modeling, corresponding to a modest 0.70 log_10_ increase in average chronic viremia relative to controls that approached statistical significance (P = 0.0503). This was accompanied by a nonsignificant trend towards lower memory CD4 T cell counts in KIR3DL01-depleted animals. Prior studies examining total NK cell depletion in SIV infection have yielded mixed results. In rhesus macaques, antibody-mediated blockade of IL-15 to deplete total NK cells had little to no effect on viral loads when depletion was initiated either prior to infection or during chronic infection [65]. In contrast, other studies have observed a significant increase in acute peak viremia following anti-IL-15-mediated total NK cell depletion [66]. Furthermore, in non-pathogenic SIV infection of African green monkeys, anti-IL-15-mediated NK cell depletion resulted in increased viral loads during chronic infection [67].

Our findings demonstrate that the targeted depletion of KIR3DL01+ NK cells results in a modest increase in chronic phase viremia (0.7 log_10_, p = 0.0503), suggesting that this subset plays a contributory role in limiting chronic SIV replication. In macaques, KIR3DL01 is highly prevalent, polymorphic, and functionally engages Mamu-Bw4 ligands [20,22,31]. In our study, four to five animals in each group expressed *Mamu-Bw4* alleles previously identified as KIR3DL01 ligands [31]. While not all Mamu-Bw4 alleles expressed in the cohort have been experimentally confirmed to interact with KIR3DL01, all animals encoded at least two major *Mamu-Bw4* alleles that could potentially serve as KIR3DL01 ligands. The phenotypic profile of KIR3DL01+ NK cells revealed that this subset is predominantly CD16+ CD56-, with lower enrichment for immature NK cell subsets. This distribution mirrors human NK cell biology, where KIR expression is generally limited to mature CD56dim NK cells [68]. In humans, the pairings of high-expressing KIR3DL1 allotypes with high affinity HLA-Bw4 I80 ligands generates highly licensed NK cells with enhanced ability to recognize Nef-mediated MHC I downmodulation [30]. Accordingly, these combinations are strongly associated with delayed progression to AIDS [29]. Our data suggest a parallel mechanism in nonhuman primates, where the targeted loss of this mature, licensed cell subset removed a highly primed NK effector population, potentially contributing to the modest increases in chronic viral load.

Furthermore, we have previously shown that KIR3DL01 is highly sensitive to the sequence of the MHC I-bound peptide, particularly to negatively charged amino acids at peptide position 8 (P8) [69]. This sensitivity is likely mediated by a negatively charged glutamic acid at residue 285 of KIR3DL01. In contrast, other Bw4-binding KIRs encode either a positively charged lysine or an uncharged glutamine at this position [31,37], rendering them less susceptible to P8 charge variations [69]. Based on crystal structures of human KIR3DL1 in complex with HLA-Bw4, this KIR position comes into close contact with P8 of the bound peptide [70]. This interaction suggests that KIR3DL01 engagement is vulnerable to disruption by SIV-induced shifts in MHC I-presented peptides, potentially triggering loss of inhibitory signaling and facilitating NK cell-mediated killing. Consequently, the targeted loss of this licensed and peptide-sensitive NK cell effector population offers a potential explanation for the modest elevation in chronic viremia observed in our cohort.

While KIRs are classically expressed on NK cells, we also observed a small population of CD8 T cells expressing KIR3DL01 (mean: 4.5%), which were similarly depleted in AAV-treated animals. KIR+ CD8 T cells have been described previously, both in rhesus macaques [31,34] and in humans [71–73]. Historically, this subset has been characterized as a terminally differentiated memory CD8 T cell population, accumulating during chronic untreated HIV infection and displaying diminished responsiveness to TCR or HIV peptide stimulation [71,74]. While these features are consistent with immune exhaustion and limited antiviral effector function, evidence suggests that KIR expression on CD8 T cells may instead confer some protection against activation-induced cell death [75,76], potentially explaining their accumulation in chronic viral infections and autoimmunity [73]. Beyond classical cytolytic functions, CD8 T cells can also exert non-cytolytic suppression of HIV replication, particularly by inducing quiescence in infected CD4 T cells via the induction of WNT and TGF-β signaling pathways, resulting in transcriptional silencing of the provirus [77,78]. Thus, we cannot exclude the possibility that the loss of KIR3DL01+ CD8 T cells contributed to the observed increase in chronic viremia.

Although NK cells play a well-established role in the control of CMV and other herpesvirus infections [46,47], we did not observe rhCMV reactivation in plasma following KIR3DL01+ NK cell depletion. Reactivation was detected in multiple animals only during chronic SIV infection (Weeks 24–28 PI), with no significant differences between KIR3DL01-depleted and control groups. This aligns with prior reports showing that total NK cell depletion by anti-IL-15 administration does not alter rhCMV reactivation in the lung [65]. Total NK cell depletion can exacerbate the reactivation of other γ-herpesvirus and associated lymphoma incidence in rhesus macaques [65]. Similarly in humans, specific KIR haplotypes exhibit different susceptibilities to γ-herpesvirus-associated tumors [79] and to CMV reactivation in transplant settings [80]. Despite these associations, our data suggest that targeted depletion of the KIR3DL01+ subset alone is insufficient to drive herpesvirus reactivation prior to chronic SIV infection.

While we previously observed a significant upregulation of KIR3DL01 on GALT NK cells during SIV infection [81], we did not detect this shift within the control group of the current study. The NKVFS1 antibody recognizes KIR3DL01 allotypes containing an aspartic acid at position 233 (D233), but does not bind allotypes containing a histidine (H233) [31]. To ensure complete depletion, all AAV-treated animals were D233 homozygous. However, three control animals were D233/H233 heterozygous (KIR3DL01*002:01, rhbo12, r20058, and r21033), possibly leading to an underestimation of total KIR3DL01 expression in those control group animals. Additional factors, including sample size of the control group, MHC I haplotypes, and differences in baseline KIR3DL01 expression may also have contributed to the lack of detectable KIR3DL01 upregulation in GALT.

Assessing NK cell phenotypes, SIV infection induced robust activation, proliferation, and maturation across both groups. During acute infection, animals exhibited a marked expansion of activated CD69+ NK cells in blood at week 2 PI, coinciding with peak viremia, which persisted throughout the chronic phase. Concurrently, we observed a transient expansion of Ki67+ NK cells at week 3 PI before returning to baseline after week 8 PI, displaying similar kinetics across blood, GALT, and lymph nodes. These activation and proliferation dynamics align with prior reports of acute SIV infection [81–84]. In parallel, immature, cytokine-producing CD16-CD56+ NK cells declined in the periphery during weeks 2–3 PI, followed by an accumulation of intermediate CD16-CD56- DN NK cells [38–40,85]. By the chronic phase, we observed the enrichment of mature, cytolytic CD16+ CD56- NK cells in lymph nodes [51,82], a well-documented phenotypic shift likely driven by sustained viral antigen exposure at sites of viral replication [86]. Additionally, AAV-treated animals exhibited a reduction in NKp46 expression on LN NK cells during chronic infection. A previous study noted increased NKp46 expression on CD16-CD56+ and DN NK cells [39]. Thus, this decrease in NKp46 expression likely reflects the loss of CD16-CD56+ NK cells in the LN during chronic infection. Finally, we observed a significant expansion of NKG2D+ NK cells in blood during chronic SIV infection. This contrasts with chronic HIV infection, where viral evasion by Nef-mediated downmodulation or indirect proteolytic shedding of NKG2D ligands (MICA/B and ULBP1–6) induces NKG2D downregulation and NK cell dysfunction [87–92]. The sustained expansion observed here may suggest species- or virus-specific differences, potentially reflecting differential modulation of stress-induced ligands by SIV or distinct responsiveness of rhesus macaque NK cells to sustained NKG2D signaling.

While previous studies assessed total NK cell depletion during SIV infection, the specific contributions of individual NK cell subsets have remained difficult to define. By utilizing AAV delivery of a monoclonal antibody, we successfully maintained the targeted depletion of KIR3DL01+ NK cells for over 40 weeks in peripheral blood and tissue. After SIV challenge, loss of this NK cell population did not alter acute peak viral loads. However, the prolonged loss of KIR3DL01+ NK cells resulted in a modest increase in SIV viremia during chronic infection. Together, these findings suggest that individual NK cell populations actively contribute to the immune-mediated reduction of viral replication during chronic infection.

## Materials and methods

### Ethics statement

All captive bred rhesus macaques (*Macaca mulatta*) of Indian ancestry enrolled in this study were housed at the Wisconsin National Primate Research Center (WNPRC) in accordance with the guidelines of the Association for the Assessment and Accreditation of Laboratory Animal Care and the University of Wisconsin Research Animal Resources and Compliance Unit (UW-RARC). All experimental procedures were approved by Institutional Animal Care and Use Committee (IACUC) protocol G005496 and conducted in accordance with the Guide for the Care and Use of Laboratory Animals [93]. Water was continuously available, and animals were provided commercial monkey chow twice daily and fresh produce once daily. To minimize distress during experimental procedures (blood collection, lymph node and colon biopsies, and SIV challenge), animals were sedated with ketamine HCl and dexmedetomidine prior to procedures and were monitored twice daily by veterinary staff. The animals were socially housed in pairs or groups whenever possible.

### Humane SIV endpoint criteria

SIV-infected rhesus macaques were monitored by WNPRC veterinary staff. Animals exhibiting clinical indicators of advanced disease, including ≥20% weight loss from baseline, treatment-refractory opportunistic infections, chronic gastrointestinal disease, inappetence, and/or neurological abnormalities, were euthanized with sodium pentobarbital and ketamine prior to week 36 post-infection. All remaining animals were electively euthanized at or beyond week 36 post-infection.

### Animal screening and NKVFS1 clone specificity

Twelve rhesus macaques were used in this study. To identify prospective KIR3DL01 + animals, PBMCs were screened for KIR3DL01 expression by flow cytometry using the pan-human KIR2D antibody NKVFS1 (Miltenyi Biotec, Bergisch Gladbach, Germany) previously shown to cross-react with rhesus macaque KIR3DL01 allotypes containing an aspartic acid at position 233 (D233) [31]. To confirm NKVFS1 specificity, Jurkat cells transduced with 25 FLAG-tagged rhesus macaque KIR constructs [37,94] were stained with a near-IR viability dye (Invitrogen, Waltham, MA), followed by NKVFS1-PE (Miltenyi Biotec) and anti-FLAG tag-BV421 (L5, BioLegend, San Diego, CA). Samples were acquired on a BD FACS Symphony flow cytometer (BD Biosciences, Franklin Lakes, NJ) and analyzed using FlowJo 10.10 software (BD Biosciences) (S1 Fig).

### KIR- and MHC I-genotyping

*MHC I* genotyping was performed by deep sequencing of exon 2 amplicons on an Illumina MiSeq instrument (Illumina, San Diego, CA) [95,96]. The *MHC I* genotypes for all study animals are summarized in S1 Table. Animals expressing *MHC I* alleles associated with spontaneous control of SIV infection (Mamu-B*008 and Mamu-B*017) were excluded from the study [32,33]. All study animals were *KIR* genotyped by PacBio long-read sequencing (Pacific Biosciences, Menlo Park, CA) of full-length cDNA amplicons as described previously [81,97,98]. *KIR* genotypes are summarized in S2 Table. GenBank accession numbers for newly identified *KIR* alleles are summarized in S4 Table.

### Amplification and cloning of NKVFS1 heavy and light chain variable regions

Heavy (V_H_) and light (V_L_) chain variable regions were amplified from NKVFS1 mouse hybridoma cells as previously described [99]. Total RNA was extracted from hybridoma cells using the NucleoSpin RNA purification kit (Macherey-Nagel, Düren, Germany), and first-strand cDNA was synthesized using SuperScript IV Reverse Transcriptase (Thermo Fisher Scientific, Waltham, MA) with 3’ J_H_ or 3’ J_κ_ primer mixes. V_H_ and V_L_ were amplified by touchdown PCR using PfuUltra II Fusion Polymerase (Agilent Technologies, Santa Clara, CA) using 5’ V_H_ or 5’ V_L_ primer pools paired with 3’ J_H_ or 3’ J_κ_ primer pools, respectively. PCR products of expected size (~500 bp) were gel purified, cloned into the Zero Blunt TOPO vector (Thermo Fisher Scientific), transformed into TOP10F’ *E. coli*, purified as plasmids, and Sanger sequenced. V_H_ and V_L_ sequences were verified by NCBI BLAST to exclude endogenous hybridoma-derived transcripts. Native or codon-optimized V_H_ and V_L_ sequences were synthesized as gBlocks (Genscript, Piscataway, NJ), subcloned in frame with rhesus macaque IgG1 constant regions, and cloned into AAV expression constructs to generate a “simianized” NKVFS1. The final construct, consisting of the native heavy chain and codon-optimized light chain, was selected based on expression efficiency and the produced antibody was confirmed to bind to KIR3DL01-transduced Jurkat cells and primary KIR3DL01+ NK cells by flow cytometry.

### AAV vector design

AAV vectors were designed and produced as outlined previously [54]. The rhesus macaque IgG1 version of NKVFS1 was expressed under control of a CMV enhancer/chicken β-actin promoter. The heavy and light chain sequences were separated by a P2A ribosomal skip sequence and a furin cleavage site. LS mutations (M428L and N434S) were introduced into the IgG1 heavy chain to extend *in vivo* half-life of the antibody through increased affinity to the neonatal Fc receptor [41,42]. A rhodopsin tag was appended to the heavy chain C-terminus for quantification by ELISA. Three tandem miRNA-142T sites were included in the 3’ UTR to limit off-target expression in antigen-presenting cells [43,44]. AAV serotype 9 (AAV9) vectors packaged with the NKVFS1 expression construct were produced by transfecting HEK293 cells with the recombinant AAV vector, AAV9 packaging, and Adenovirus helper plasmids as described previously [100,101]. AAV9 particles were purified from lysates by density gradient centrifugation, quantified by genome copy number (gc/ml) by digital droplet PCR, verified for purity by silver-stained SDS-PAGE, aliquoted, and stored at -80°C.

### AAV vector administration

Animals were prescreened for neutralizing antibodies against AAV9 prior to group assignment, with AAV9-seronegative animals selected for AAV administration (S3 Table) [55,102]. Six KIR3DL01+ rhesus macaques received the NKVFS1-AAV9 vector by intramuscular injection at a dose of 3.0x10^12^ genome copies/kg diluted in PBS and administered using tuberculin syringes. The inoculum was distributed across six sites (two deltoid, two quadriceps, and two biceps, 0.5 ml per site) to maximize muscle cell transduction. A control group of six KIR3DL01+ animals received mock injections with PBS at the same sites.

### NKVFS1 serum concentrations

NKVFS1 containing a C-terminal rhodopsin tag (C9) was produced by transfecting Expi293 cells with the NKVFS1-AAV antibody expression plasmid using the ExpiFectamine 293 transfection kit (Thermo Fisher Scientific), according to the manufacturer’s protocol. Antibodies were purified from cell supernatants on rProtein A GraviTrap columns (Cytiva Life Sciences, Marlborough, MA) [103]. NKVFS1-C9 was used to generate a two-fold dilution standard calibration curve ranging from 0.625 to 20 ng/ml.

For antibody expression measurements, blood was collected in serum separator tubes (SST), centrifuged at 2500 rpm for 25 minutes, and serum was aliquoted and stored at -80°C. ELISAs to detect C9-tagged antibodies in serum were performed as previously described [54], using Reacti-Bind plates (Thermo Fisher Scientific) coated with an anti-rhodopsin antibody (clone 1D4, Sigma Aldrich, St. Louis, MO). Reciprocal two-fold serum dilutions and NKVFS1 antibody standards were incubated at 37 °C followed by detection with HRP-conjugated anti-monkey IgG (1:6000 dilution) (SB108a, Southern Biotech, Birmingham, AL) and developed in SureBlue tetramethylbenzidine (TMB) substrate (LGC Clinical Diagnostics, Milford, MA). The reaction was stopped with an equal volume of 1M H_2_SO_4_, and 450 nm absorbance was quantified on a Victor X4 plate reader (PerkinElmer, Waltham, MA).

### Anti-drug antibody quantification

ADA responses were measured as previously described [54]. Total IgG was purified from heat-inactivated serum using rProtein A Sepharose Fast Flow beads (Cytiva Life Sciences) and biotinylated with ChromaLINK biotin (Vector Laboratories, Newark, CA). As a negative control, equal volumes of pre-AAV serum from all study animals were pooled. As a positive standard, 40 μg/ml of polyclonal rabbit anti-mouse IgG (Jackson ImmunoResearch, West Grove, PA) was spiked into a separate aliquot of pre-AAV pooled serum. IgG from control and experimental samples were isolated and biotinylated in parallel.

Reacti-Bind plates were coated overnight at 4°C with 2 μg/ml of NKVFS1-C9 or polyclonal mouse IgG (Jackson ImmunoResearch). Two-fold serial dilutions of biotinylated IgG (1:100 to 1:3200) were added to NKVFS1-C9 coated wells, while control and standard samples were added to mouse IgG-coated wells and incubated for one hour at 37°C. After washing, bound biotinylated IgG was detected using Streptavidin-HRP conjugate (1:10000 dilution) (N504, Thermo Fisher Scientific), followed by TMB substrate development, stopped with 1M H_2_SO_4_, and read at 450 nm on a Victor CX4 plate reader. ADA titers were quantified as equivalent IgG concentrations relative to the binding of the biotinylated rabbit anti-mouse IgG standard to polyclonal mouse IgG wells.

### SIV challenge

Five weeks after AAV9 administration, animals were challenged intrarectally (IR) with SIVmac239 (3,000 TCID50) as described previously [104]. Prior to challenge, SIVmac239 stocks were diluted to 3,000 TCID50 in 1 ml RPMI (Cytiva Life Sciences) and loaded into syringes. Animals were anesthetized with their pelvises elevated, and the viral inoculum was slowly administered across the rectal mucosa.

### SIV plasma viral load measurements

Plasma SIV RNA loads were measured by quantitative real-time/digital RT-PCR using primers amplifying a conserved region of SIV *gag*, with a limit of detection of 15 copies/ml, as previously described [105,106].

### rhCMV plasma viral load measurements

To assess potential reactivation of rhCMV following KIR3DL01+ NK cell depletion or SIV infection, plasma rhCMV DNA was quantified by real-time PCR targeting rhCMV UL54, with a limit of detection of 325 DNA copies/ml, as outlined previously [48]. Briefly, DNA was isolated from plasma using the Zymo Quick DNA Miniprep Kit (Zymo Research, Irvine, CA). rhCMV DNA was then quantified by qPCR using rhCMV UL54-specific primers and TaqMan Fast Advanced Master Mix (Thermo Fisher Scientific).

### Lymphocyte isolation

#### i. Peripheral blood.

Peripheral blood was collected in EDTA-treated tubes. Whole blood was layered onto Ficoll-Paque PREMIUM (Cytiva Life Sciences) and centrifuged at 2000 rpm for 25 minutes without brake. Plasma was isolated from the top layer and centrifuged at 2500 rpm for 25 minutes to remove residual cell contamination. Plasma was aliquoted and stored at -80°C for viral load measurements. PBMCs were isolated from the plasma/Ficoll interface, washed in RPMI, and treated with ACK red blood cell lysing buffer (Quality Biological, Gaithersburg, MD). Cell yield was quantified using a Luna II automated cell counter (Logos Biosystems, Anyang-si, South Korea). PBMCs were aliquoted for flow cytometric analysis and cryopreservation.

#### ii. Lymph nodes.

Lymphocytes were isolated from axillary or inguinal lymph node biopsies. Surrounding fat was trimmed from the lymph nodes using sterile scalpels, and lymphocytes were released from the tissue by mechanical disruption. Cells were resuspended in RPMI and passed through 100 μm nylon filters. Following filtration, lymphocytes were washed in RPMI and treated with ACK red blood cell lysing buffer. Final cell yield was quantified using a Luna II automated cell counter and aliquoted for flow cytometric analysis and cryopreservation.

#### iii. Gut-associated lymphoid tissue.

Lymphocytes were isolated from colon biopsies and whole colon sections at necropsy, as previously described [81,107]. Colon pinch biopsies were collected from 10-12 sites and incubated by shaking at 37°C in Hank’s Balanced Salt Solution (HBSS) supplemented with 1 mM EDTA for 30 minutes. Following washing, biopsies were digested for one hour in RPMI containing 10% fetal bovine serum (FBS) (R10) supplemented with 0.5 mg/ml type II collagenase (Sigma-Aldrich), with mixing by pipetting halfway through incubation. Tissues were then washed twice and passed through 100 μm nylon filters. The cell suspension was layered over a discontinuous 35/60% isotonic Percoll gradient (Sigma-Aldrich) and centrifuged at 1000 xg for 30 minutes at 4°C with brake off. Lymphocytes were collected from the interface between 35% and 60% Percoll layers, washed in RPMI, counted to confirm yield, and aliquoted for flow cytometry. Whole colon sections collected at necropsy were processed similarly, except tissues were finely minced into ~1 mm fragments prior to collagenase digestion and were not pre-treated with HBSS + EDTA. Following filtration, the cell suspension was resuspended in 40% isotonic Percoll and layered over 80% Percoll for lymphocyte isolation.

### Flow cytometry

All flow cytometry data were collected on a BD FACS Symphony (BD Biosciences) and analysis was done using FlowJo 10.10 software (BD Biosciences). All the antibodies listed are murine origin unless specified otherwise.

#### i. NK cell phenotyping panel.

Lymphocytes were incubated with 0.5 μg of BV421-conjugated Mamu-A1*002 tetramers folded with Gag 71–79 GY9 peptide (NIH tetramer core, Emory University, Atlanta GA) for 1 hour at 37 °C in R10 medium to identify KIR3DL05+ NK cells. Cells were then washed twice in PBS + 2% FBS (FACS buffer) and stained with a near-IR live/dead dye (Invitrogen) for 20 minutes at room temperature. After two additional washes in FACS buffer, cells were incubated for 30 minutes at 4°C with the following antibodies: anti-CD45 BV786 (D058-1283, BD Biosciences), anti-CD3 Alexa Fluor 700 (SP34-2, BD Biosciences), anti-CD20 Alexa Fluor 700 (2H7, BD Biosciences), anti-CD14 Alexa Fluor 700 (M5E2, BD Biosciences), anti-CD8 BV711 (RPA-T8, BD Biosciences), anti-CD16 BV605 (3G8, BD Biosciences), anti-NKG2AC PECy7 (Z199, Beckman Coulter), anti-CD56 PE-CF594 (B159, BD Biosciences), anti-KIR3DL01 PE (NKVFS1, Miltenyi Biotec), anti-CD69 BV510 (FN50, BD Biosciences), anti-DNAM-1 BUV563 (DX11, BD Biosciences), anti-NKp46 PECy5 (BAB281, Beckman Coulter), and anti-NKG2D APC (BAT221, Miltenyi Biotec). Parallel samples were stained with the same antibody panel except that the antibodies against the following markers (CD69, DNAM-1, NKp46, and NKG2D) were replaced with matched isotypes for gating control: BV510 mouse IgG1 isotype (KLH, BD Biosciences), BUV563 mouse IgG1 isotype (KLH, BD Biosciences), PECy5 mouse IgG1 isotype (MOPC-21, BD Biosciences), and APC mouse IgG1 isotype (MOPC-21, BD Biosciences). Following surface staining, cells were washed twice in FACS buffer and fixed in 2% paraformaldehyde (PFA) for 15 minutes. Fixed cells were then washed twice and permeabilized in Medium B FIX & PERM Solution (Thermo Fisher Scientific) before intracellular staining with anti-Ki67 BUV395 (B56, BD Biosciences) or a corresponding BUV395 mouse IgG1 isotype (KLH, BD Biosciences). After two washes in FACS buffer, cells were fixed in 2% PFA and analyzed by flow cytometry. Representative gating is shown in (S2 Fig).

#### ii. T cell panel.

Isolated PBMCs were stained with near-IR live/dead dye (Invitrogen) for 20 minutes at room temperature, washed twice with FACS, and incubated for 30 minutes at 4°C with the following antibodies: anti-NKG2AC PECy7 (Z199, Beckman Coulter), anti-CD16 Pacific Blue (3G8, BD Biosciences), anti-CD20 BUV395 (2H7, BD Biosciences), anti-CD3 PE-CF594 (SP34-2, BD Biosciences), anti-CD4 APC (OKT4, eBioscience), anti-CD8 BV711 (RPA-T8, BD Biosciences), anti-CD95 PECy5 (DX2, BD Biosciences), anti-CCR7 FITC (150503, BD Biosciences), and anti-CD28 PE (CD28.2, eBioscience). Cells were subsequently washed twice with FACS buffer and fixed in 2% PFA. Samples were analyzed by flow cytometry. Representative gating is shown in (S4 Fig).

#### iii. KIR3DL01 depletion panel.

Lymphocytes were stained with near-IR live/dead dye (Invitrogen) for 20 minutes at room temperature, washed twice with FACS buffer, and incubated for 30 minutes at 4°C with the following antibodies: anti-CD45 BV786 (D058-1283, BD Biosciences), anti-CD3 PE-CF594 (SP34-2, BD Biosciences), anti-CD20 BUV395 (2H7, BD Biosciences), anti-CD14 Alexa Fluor 700 (M5E2, BD Biosciences), anti-CD8 BV711 (RPA-T8, BD Biosciences), anti-NKG2AC PECy7 (Z199, Beckman Coulter), anti-KIR3DL01 PE (NKVFS1, Miltenyi Biotec), and anti-rhodopsin Alexa Fluor 647 (1D4, Santa Cruz Biotechnology, Dallas, TX). Cells were subsequently washed twice with FACS buffer and fixed in 2% paraformaldehyde (PFA). Samples were analyzed by flow cytometry. Representative gating is shown in (S5 Fig).

#### iv. Absolute cell quantification.

Whole blood (50 μl) was stained for 15 minutes at room temperature in Trucount tubes (BD Biosciences) with the following antibodies: anti-CD45 PerCP (D058-1283, BD Biosciences), anti-CD3 PE-CF594 (SP34-2, BD Biosciences), anti-CD20 BUV395 (2H7, BD Biosciences), anti-CD8 BV711 (RPA-T8, BD Biosciences), anti-CD4 APC (OKT4, eBioscience, San Diego, CA), anti-NKG2AC PECy7 (Z199, Beckman Coulter, Brea, CA), and anti-KIR3DL01 PE (NKVFS1, Miltenyi Biotec). Red blood cells were lysed, and samples were fixed with 450 μl of BD FACS lysing solution (BD Biosciences). Samples were analyzed by flow cytometry. Representative gating is shown in (S6 Fig). Bead standard events were identified by size gating. Absolute cell counts (cells/μl) were calculated as (cell events/ bead events) x (bead standard/ 50 μl).

### Statistical analysis

Statistical analyses were conducted in RStudio using R (version 4.5.1). Longitudinal immune cell phenotypes were analyzed via linear mixed-effects models (LMMs) employing the lme4 package. Absolute PBMC immune cell counts were log_10_-transformed, while tissue NK cell phenotypes were analyzed as percent positive to meet model assumptions. For all LMMs, individual animal was included as a random intercept to account for repeated measures, and time was modeled as a categorical fixed effect encompassing all available timepoints. For within-group analysis, baseline values were defined as per-animal averages over predefined windows (pre-AAV, weeks -8 to -5 and pre-SIV weeks -3 to 0). Separate LMMs were fitted for AAV-treated and control animals to contrast post-treatment timepoints against baselines independently. Between group differences were evaluated using LMMs with a Group x Timepoint interaction, followed by pairwise contrasts at each timepoint. Model p-values were obtained using lmerTest, marginal means and contrasts were estimated via emmeans, and all p-values were adjusted using the Benjamini-Hochberg false discovery rate (FDR) method.

Co-expression profiles of KIR-defined NK cell subsets were analyzed in GraphPad Prism version 10.5 (GraphPad Software, San Diego, CA) using LMMs. To account for repeated measures, the two pre-administration timepoints were treated as replicates, allowing for comparison of differences across the distinct NK cell subsets.

Longitudinal SIV plasma viral loads were log_10_-transformed and analyzed using the identical LMM pipeline as described above to evaluate between-group differences at specific timepoints. To compare aggregate chronic phase SIV viremia, the mean of the log10-transformed plasma viral loads from week 8 to week 35 post infection was calculated for each animal, and differences between groups were assessed using an unpaired t-test in GraphPad Prism.

rhCMV detection was assessed as a binomial distribution (0 = below limit of detection, 1 = detectable) and analyzed using a binomial generalized linear mixed model (GLMM) using the lme4 package, with individual animal included as a random intercept to account for repeated measurements. P-values were extracted from the model summary and adjusted for multiple comparisons using FDR correction. Survival analysis was performed in GraphPad Prism using Kaplan-Meier curves and the Gehan-Breslow-Wilcoxon test.

## Supporting information

S1 Fig Binding specificity of the NKVFS1 antibody across 25 different rhesus macaque KIR allotypes. **(A)** Flow cytometry histogram overlay and corresponding geometric mean fluorescence intensities of NKVFS1 staining against Jurkat cells transduced with 25 distinct FLAG-tagged rhesus macaque KIR constructs representing distinct allotypes [37,94]. Cells were gated on live singlets prior to quantification. **(B)** Representative plot of FLAG-tagged KIR3DL01*013-transduced Jurkat cells co-stained for anti-FLAG-BV421 and NKVFS1-PE.
(TIF)

S2 Fig NK cell phenotyping panel gating strategy. Isolated lymphocytes were stained with A1*002 Gag GY9 tetramer (KIR3DL05), followed by a Near IR live/dead dye, and antibodies against CD45, CD3, CD20, CD14, CD8α, CD16, NKG2AC, CD56, KIR3DL01, CD69, DNAM-1, NKp46, and NKG2D. Cells were fixed with PFA and intracellularly stained in Medium B using an anti-Ki67 antibody. Parallel samples were stained with the same antibody panel, substituting matched isotype/concentration controls for CD69, DNAM1, NKp46, NKG2D, and Ki67 to define gating thresholds. NK cells were defined as CD45+, CD3-14-20-, NKG2AC+CD8α+.
(TIF)

S3 Fig Baseline NK cell phenotypes of KIR defined NK cell subsets. Frequencies of NK cell populations were evaluated in PBMCs three weeks before and immediately prior to AAV administration. Flow cytometric analysis assessed the frequencies of **(A)** mature CD16+ CD56-, **(B)** immature CD16-CD56+, and **(C)** intermediate CD16-CD56- NK cells, as well as **(D)** the expression of the activating receptor NKG2D. Phenotypes were compared across KIR3DL01+ single-positive (L01+ L05-, orange), KIR3DL05+ single-positive (L01- L05+, green) and KIR-negative (L01- L05-, grey). Horizontal lines denote the mean. Asterisks indicate significant differences by linear mixed-effects modeling, incorporating two pre-administration timepoints accounting for repeated measures (* p < 0.05 and ** p < 0.01).
(TIF)

S4 Fig T cell phenotyping panel gating strategy. Isolated PBMCs were stained with Near IR live/dead dye, followed by antibodies against NKG2AC, CD16, CD20, CD3, CD4, CD8, CD95, CD28, and CCR7, followed by fixation with PFA. CD3+ CD8+ and CD3+ CD4+ were classified as naïve (CD95-) or memory (CD95+). CD95+ memory subsets were further distinguished as effector memory (CD28-), transitional memory (CCR7-CD28+), or central memory (CCR7+ CD28+).
(TIF)

S5 Fig KIR3DL01 depletion panel gating strategy. Isolated lymphocytes were stained with Near IR live/dead dye, followed by antibodies against CD45, CD3, CD20, CD14, CD8α, NKG2AC, KIR3DL01, and rhodopsin (C9), followed by fixation with PFA. CD45+ lymphocytes were classified as CD20+, CD3+, or CD3–20-. CD3+ T cells were differentiated as CD8- or CD8+ and analyzed for KIR3DL01 expression. CD3–20- population was further subdivided into monocytes (CD14+) or NK cells (CD8α+NKG2AC+). NK cells and CD8 T cells evaluated for KIR3DL01 expression.
(TIF)

S6 Fig Trucount absolute cell counts quantification strategy. Whole blood (50 μl) was stained in Trucount tubes with the bead standard and antibodies against CD45, CD3, CD20, CD8, CD4, NKG2AC, and KIR3DL01, and then fixed using BD FACS lysing solution. CD45+ lymphocytes were classified as CD20+, CD3+, or CD20- CD3-. NK cells defined as CD3–20-, CD8α+NKG2AC+. CD3+ T cells were further subdivided into CD4+ or CD8+ subsets. Bead standard events were determined by size gating. Absolute cell counts (cells/μl) were calculated as (cell events/ bead events) x (bead standard/ 50 μl whole blood).
(TIF)

S7 Fig Direct detection of AAV delivered NKVFS1 binding to KIR3DL01+ NK cells by rhodopsin C9 staining. Isolated lymphocytes were stained with the KIR3DL01 depletion flow cytometry panel as described in S5 Fig. Cell surface expression of KIR3DL01 was assessed against binding of the AAV-vectored NKVFS1 antibody, detected via its rhodopsin C9 tag. Representative staining for the AAV-treated group is shown on day of AAV administration, one week post-AAV administration, and four weeks post-SIVmac239 infection.
(TIF)

S8 Fig T cell dynamics following SIV infection. Longitudinal changes in total T cells **(A)**, CD8 T cells **(B)**, effector memory CD8 T cells **(C)**, CD4 T cells **(D)**, and memory CD4 T cells **(E)** in blood. Lines indicate geometric means ± SD of log_10_-transformed cell counts per group (control: blue and AAV: red). Absolute counts of total CD3+ and CD8+ T cells were quantified from Trucount whole blood staining. Counts of effector memory CD8 (CD3+ CD8+ CD95+ CD28-), CD4, and memory CD4 (CD3+ CD4+ CD95+) subsets were calculated by multiplying their frequencies by total T cell counts at each timepoint.
(TIF)

S1 Table MHC genotypes of study animals. *MHC I* alleles of *Mamu-A* and *-B* alleles present in study animals. Animal IDs are colored-coded by treatment group: AAV (red) and control (blue). Mamu-Bw4 ligands previously identified to be ligands for KIR3DL01 are bolded [31,37].
(DOCX)

S2 Table KIR alleles present in study animals. Animal IDs are colored-coded by treatment group: AAV (red) and control (blue). The suffix _nov1 designates novel *KIR* alleles identified in this study. KIR3DL11*008N_nov1 is a pseudogene containing a frameshift mutation. *S06-L07_rec1* represents a novel recombination event, retaining the D0 and D1 domains of *KIR3DS06* and D2 through transmembrane inhibitory domains of *KIR3DL07*.
(DOCX)

S3 Table Demographic information for study animals. Ages and weights are at the time of AAV administration. Percentage of KIR3DL01+ NK cells are average from pre-AAV timepoints (weeks -8, -6 and -5 prior to SIV infection).
(DOCX)

S4 Table GenBank accession numbers for newly identified *KIR* alleles. (DOCX)

S1 Data Numerical data for all charts and graphs. This file contains the raw underlying data used to generate the figures and statistical analysis presented in this study.
(XLSX)
