# Supplementary material for: Long-term depletion of KIR3DL01+ NK cells by adeno-associated viral-vectored antibody delivery alters chronic SIV infection
Source: PLoS Pathog. 2026 Aug 5;22(8):e1014508. doi: 10.1371/journal.ppat.1014508 (PMC13456248; doi:10.1371/journal.ppat.1014508)
Supplement: Supplementary file 1 [file ppat.1014508.s009.docx]

| **AAV group** | **r20066** | **rh3074** | **rh3099** | **rhbn55** | **rhbn96** | **rh3084** |
| --- | --- | --- | --- | --- | --- | --- |
| **Mamu-A - Major** | A1*001 | A1*008 | A1*004 | A1*007 | A1*004 | A1*002 |
|  | A1*028 | A1*011 | A1*007 | A1*008 | A1*023 | A1*019 |
|  |  |  |  |  |  |  |
| **Mamu-A - Minor** | A2*05 | A2*05 | A2*05 | A2*05 | A4*14 | A2*05 |
|  | A4*14 | A3*13 | A4*14 | A3*13 |  | A3*13 |
|  |  | A6*01 | A6*01 | A6*01 |  | A4*14 |
|  |  |  |  |  |  |  |
| **Mamu-B - Major** | B*012 | B*005 | B*012 | B*001 | B*001 | B*012 |
|  | B*030 | B*015 | **B*022** | B*006 | **B*007** | B*019 |
|  | **B*043** | B*044 | B*030 | **B*007** | B*030 | B*024 |
|  | B*082 | B*052 | B*031 | B*030 | **B*041** | B*030 |
|  |  | B*055 | **B*041** | B*047/B*071 | B*048 | B*082 |
|  |  | **B*058** | B*048 |  |  |  |
|  |  |  | B*064 |  |  |  |
|  |  |  |  |  |  |  |
| **Mamu-B - Minor** | B*035/B*049 | B*054 | B*035/B*049 | B*035/B*049 | B*035/B*049 | B*035/B*049 |
|  | B*046 | B*060 | B*053 | B*051 | B*054 | B*046 |
|  | B*060 | B*063 | B*054 | B*057 | B*060 | B*051 |
|  | B*098 | B*072 | B*057 | B*063 | B*063 | B*057 |
|  | B*109 | B*098 | B*063 | B*072 | B*072 | B*072 |
|  |  | B*109 | B*097 | B*109 | B*109 | B*098 |
|  |  | B*134 | B*098 | B*188 | B*134 | B*109 |
|  |  | B*178 | B*109 |  | B*188 |  |
|  |  |  | B*134 |  |  |  |
|  |  |  |  |  |  |  |
| **Control group** | **rhbn52** | **rhbo12** | **rh3083** | **r19016** | **r20058** | **r21033** |
| **Mamu-A - Major** | A1*001 | A1*004 | A1*002 | A1*004 | A1*001 | A1*001 |
|  | A1*004 |  |  | A1*023 |  | A1*008 |
|  |  |  |  |  |  |  |
| **Mamu-A - Minor** | A3*13 | A4*14 | A3*13 | A4*14 | A2*05 | A2*05 |
|  | A4*14 |  |  |  |  | A3*13 |
|  |  |  |  |  |  |  |
|  |  |  |  |  |  |  |
| **Mamu-B - Major** | B*019 | B*005 | B*005 | B*012 | B*030 | B*012 |
|  | B*024 | B*012 | B*012 | **B*022** | B*031 | B*015 |
|  | **B*041** | B*015 | B*015 | B*027 | **B*043** | B*030 |
|  | B*048 | **B*022** | B*030 | B*030 | B*048 | B*068 |
|  | B*082 | B*030 | B*082 | B*031 | B*073 | B*082 |
|  |  | B*031 |  | **B*043** |  |  |
|  |  |  |  |  |  |  |
|  |  |  |  |  |  |  |
| **Mamu-B - Minor** | B*035/B*049 | B*035/B*049 | B*035/B*049 | B*035/B*049 | B*054 | B*035/B*049 |
|  | B*046 | B*057 | B*057 | B*053 | B*057 | B*046 |
|  | B*054 | B*060 | B*060 | B*057 | B*060 | B*051 |
|  | B*057 | B*063 | B*072 | B*060 | B*072 | B*057 |
|  | B*063 | B*072 | B*098 | B*063 | B*097 | B*060 |
|  | B*072 | B*098 | B*109 | B*070 | B*109 | B*098 |
|  | B*109 | B*109 | B*178 | B*092 | B*134 | B*109 |
|  | B*134 | B*178 |  | B*098 |  | B*180 |
|  |  |  |  | B*109 |  |  |
