# Supplementary material for: Long-term depletion of KIR3DL01+ NK cells by adeno-associated viral-vectored antibody delivery alters chronic SIV infection
Source: PLoS Pathog. 2026 Aug 5;22(8):e1014508. doi: 10.1371/journal.ppat.1014508 (PMC13456248; doi:10.1371/journal.ppat.1014508)
Supplement: Supplementary file 2 [file ppat.1014508.s010.docx]

|  | **AAV group** | |  |  |  |  | **Control group** | |  |  |  |  |
| --- | --- | --- | --- | --- | --- | --- | --- | --- | --- | --- | --- | --- |
|  | **r20066** | **rh3074** | **rh3099** | **rhbn55** | **rhbn96** | **rh3084** | **rhbn52** | **rhbo12** | **rh3083** | **r19016** | **r20058** | **r21033** |
| **KIR1D** | *003:01 *013 | *002 |  | *001 *002 | *001 *002 | *001 *002 |  | *002 | *001 *002 | *001 *002 | *001 *002 | *001 *002 |
| **KIR2DL04** | *008:02 | *002:01 |  |  | *008:02 | *001:01 | *002:01 |  | *001:01 |  |  | *015:01 |
| **KI3DL20** | *016:02 | *001 *014 | *030R |  | *014 | *014 | *030R *059 | *014 | *001 *035 | *014 | *014 |  |
| **KIR3DL01** | *016 | *057 _nov1 | *015:01 *019:01 | *001 | *015:01 | *012 *032 | *015:01 *059 | *002:01 *013 | *012  _nov1 | *015:01 *019:01 *013  _nov1 | *002:01 *012 | *002:01 *019:01 |
| **KIR3DL02** | *009:01 | *007 *007 _nov1 | *009:01 | *004:01  _nov1 |  |  | *007 *009:01 | *004:02 |  | *009:01 |  | *004:01 |
| **KIR3DL04** | *001:01 |  |  |  |  |  |  |  |  |  |  |  |
| **KIR3DL05** | *029  _nov1 | *006:01 | *006:01 |  | *006:01 *008:01 |  | *006:01 _nov1 |  | *003 *036 |  |  | *008:04 *020 |
| **KIR3DL07** | *004 | *040 | *003 *028 |  | *018:01 *019:01 *020 |  | *040  S06-L07  _rec1 |  | *020 | *019:01 | *019:02 | *003 *025 |
| **KIR3DL08** |  | *008 | *017 |  |  |  | *008  _nov1 *035  _nov1 |  |  |  |  |  |
| **KIR3DL10** |  |  |  |  |  |  |  |  |  |  | *002:01 | *002:01 |
| **KIR3DL11** |  |  |  |  | *009 |  | *008:01N _nov1 |  |  |  | *011 | *010 |
| **KIR3DLw03** | *007 |  |  |  | *006:01 *007 | *006:01 |  |  |  |  | *007 |  |
| **KIR3DLw45** |  |  |  |  |  |  |  |  |  |  | *006 |  |
|  |  |  |  |  |  |  |  |  |  |  |  |  |
| **KIR3DS01** |  |  |  | *001:02/*002 | *004 |  |  | *003 |  |  | *005 | *001:02/*002 |
| **KIR3DS02** | *015:07 | *015:05 | *015:05 |  | *008 *015:08 |  |  |  | *009:01 *015:07 |  | *015:05 | *015:01 |
| **KIR3DS03** |  |  |  |  |  |  |  |  |  |  |  |  |
| **KIR3DS04** | *001:02 |  |  |  |  |  |  |  | *005:03  _nov1 |  | *004 _nov1 | *001:02 |
| **KIR3DS05** |  |  |  |  |  |  |  | *002:01 | *002:01 |  | *002:01_nov1 | *002:01 |
| **KIR3DS06** |  |  |  |  |  | *002:01/02 |  |  |  |  | *011:02 | *012 |
| **KIR3DSw07** |  |  |  |  |  |  |  |  | *003 |  |  |  |
| **KIR3DSw09** |  |  | *003:02 |  |  |  |  |  |  |  |  | *003:02 |
| **KIR3DSw36** |  |  |  |  |  |  |  |  | *001 |  |  |  |
| **KIR3DSw39** |  |  |  |  | *004 *005:02 |  | *001 |  |  |  |  |  |
