# Supplementary material for: Long-term depletion of KIR3DL01+ NK cells by adeno-associated viral-vectored antibody delivery alters chronic SIV infection
Source: PLoS Pathog. 2026 Aug 5;22(8):e1014508. doi: 10.1371/journal.ppat.1014508 (PMC13456248; doi:10.1371/journal.ppat.1014508)
Supplement: Supplementary file 3 [file ppat.1014508.s011.docx]

| **Animal** | **Group** | **Sex** | **Age (years)** | **Weight (kg)** | **Average % KIR3DL01+ NK cells** | **AAV9 serostatus** |
| --- | --- | --- | --- | --- | --- | --- |
| r20066 | AAV | M | 3.8 | 7.11 | 17.43 | neg |
| rh3074 | AAV | F | 6.1 | 6.23 | 4.79 | neg |
| rh3099 | AAV | M | 5.9 | 11.4 | 2.17 | neg |
| rhbn55 | AAV | M | 6 | 7.94 | 9.38 | neg |
| rhbn96 | AAV | M | 5.4 | 8.97 | 14.00 | neg |
| rh3084 | AAV | M | 7.7 | 10.75 | 43.60 | neg |
| rhbn52 | Control | F | 5.6 | 5.48 | 7.84 |  |
| rhbo12 | Control | M | 5.2 | 11.58 | 36.30 |  |
| rh3083 | Control | M | 7 | 15.36 | 21.07 |  |
| r19016 | Control | M | 5.2 | 7.96 | 16.60 |  |
| r20058 | Control | M | 3.9 | 7.85 | 12.13 |  |
| r21033 | Control | M | 3.1 | 6.52 | 1.25 |  |
