# Supplementary material for: Long-term depletion of KIR3DL01+ NK cells by adeno-associated viral-vectored antibody delivery alters chronic SIV infection
Source: PLoS Pathog. 2026 Aug 5;22(8):e1014508. doi: 10.1371/journal.ppat.1014508 (PMC13456248; doi:10.1371/journal.ppat.1014508)
Supplement: Supplementary file 4 [file ppat.1014508.s012.docx]

| **Designation** | **Accession Number** |
| --- | --- |
| KIR3DL01*012_nov1 | PZ013195 |
| KIR3DL01*013_nov1 | PZ013196 |
| KIR3DL01*057_nov1 | PZ013197 |
| KIR3DL02*004:01_nov1 | PZ013198 |
| KIR3DL02*007_nov1 | PZ013199 |
| KIR3DL05*006:01_nov1 | PZ013200 |
| KIR3DL05*029_nov1 | PZ013201 |
| KIR3DL08*008_nov1 | PZ013202 |
| KIR3DL08*035_nov1 | PZ013203 |
| KIR3DL11*008:01N_nov1 | PZ013204 |
| KIR3DS04*004_nov1 | PZ013205 |
| KIR3DS04*005:03_nov1 | PZ013206 |
| KIR3DS05*002:01_nov1 | PZ013207 |
| KIR3DS06-3DL07_rec1 | PZ013208 |
